# Determination of reference values of figure of 8 walk walk test in healthy adults

**DOI:** 10.1007/s11845-025-04276-w

**Published:** 2026-01-19

**Authors:** Zekiye Ipek Katirci Kirmaci, Demet Gözaçan Karabulut, Çağtay Maden, Gönül Elpeze, Yusuf Şinasi Kirmaci, Hatice Yildirim, Tuba Gün Kalkin

**Affiliations:** 1 Faculty of Health Sciences, Department of Physiotherapy and Rehabilitation, Gaziantep Islam Science and Technology University, Gaziantep, Türkiye; 2 Faculty of Health Science, Department of Physiotherapy and Rehabilitation, Kahramanmaras Sutcu Imam University, Kahramanmaras, Turkey

**Keywords:** Anthropometrics, Fall, Mobility, Normative values

## Abstract

**Objective:**

The Figure-8 Walk Test (F8WT) is a standardized clinical assessment used to evaluate walking performance, including straight walking and maneuvering around obstacles, as required in daily life. The aim of this study was to establish reference values for the F8WT in healthy adults.

**Methods:**

A total of 1,145 healthy Turkish adults participated in this cross-sectional study. Individuals aged ≥ 18 years who were able to walk independently without assistive devices were included. Participants with a history of neurological, musculoskeletal, or cardiopulmonary disorders affecting gait, recent lower extremity injury or surgery, or cognitive impairment were excluded. The F8WT was administered, and anthropometric as well as sociodemographic parameters were recorded.

**Results:**

The 25th, 25th–75th, and > 75th percentiles were classified as low, average, and high fall risk for both men and women based on F8WT performance. For men, < 3.06 s indicated low fall risk, 3.06–9.34 s indicated average fall risk, and 9.34–22.31 s indicated high fall risk. For women, < 2.41 s indicated low fall risk, 2.41–8.43 s indicated average fall risk, and 8.43–16.06 s indicated high fall risk. Statistically significant differences were observed between men and women in terms of age, height, body weight, and body mass index (BMI) (*p* < 0.01). In the overall sample, F8WT performance was significantly correlated with age (r = 0.575, *p* < 0.001), height (r = –0.145, *p* < 0.001), weight (r = 0.242, *p* < 0.001), and BMI (r = 0.359, *p* < 0.001).

**Conclusion:**

This study provides reference values for the F8WT according to age and sex in healthy adults. Women demonstrated shorter F8WT completion times than men, and test duration increased with advancing age.

**Clinical trial number:**

NCT06314347.

## Introduction

Physical performance tests are widely employed by physiotherapists to identify physical impairments or functional deficiencies, monitor progress during rehabilitation, and objectively document treatment outcomes [1, 2]. Commonly used assessments in this context include the Berg Balance Scale, Dynamic Gait Index, Timed Up and Go Test, 3-Meter Backward Walk Test, and the Figure-8 Walk Test (F8WT) [3, 4]. These tests not only evaluate overall physical performance but also play a crucial role in determining fall risk and balance problems, particularly among older adults and individuals with neurological or orthopedic conditions [5, 6]. Accordingly, beyond establishing their validity and reliability across different populations, the determination of normative values is of great clinical importance.

The F8WT is a standardized clinical test designed to assess advanced walking performance required in daily life. It is a functional measure that challenges individuals to demonstrate complex motor skills such as directional changes, speed regulation, and agility while walking [4]. In this test, participants are instructed to walk a figure-of-eight trajectory as quickly and as safely as possible. Thus, the F8WT evaluates not only walking speed but also agility, turning ability, and balance strategies [7].

Turning ability is essential for many daily activities, such as walking around furniture, avoiding obstacles, and navigating crowded or uneven environments. Walking on curved versus straight paths requires different gait mechanics; for example, curved walking shifts the body’s center of mass toward the inner leg, increasing its stance time compared with straight walking [8]. While traditional walking assessments (e.g., the 10-Meter Walk Test or Timed Up and Go Test) primarily focus on linear gait, the F8WT better simulates the complex walking demands encountered in real-life situations [9]. Therefore, it is increasingly used in both clinical and research contexts as a comprehensive measure of functional mobility. Compared with other standardized walking tests, the F8WT has several advantages: it is not dependent on examiner subjectivity, can be administered with minimal training, requires little time, space, or equipment, and permits the use of assistive devices [4]. Nevertheless, normative data for the F8WT across healthy populations of varying ages remain limited.

Normative values are critical in clinical practice, as they enable the comparison of an individual’s performance against population-based references, thereby guiding diagnosis, prognosis, and treatment planning [2]. Although the F8WT has demonstrated validity and reliability in several pathological conditions [10–13], its normative values for healthy adults have not yet been established. The present study therefore aimed to determine reference values for the Figure-8 Walk Test (F8WT) in healthy adults and to classify performance-based fall risk.

## Materials and methods

This cross-sectional study included healthy adults aged 18 years and older, with no known musculoskeletal, cardiac and pulmonary disease or neurological disorders, and who were not elite or professional athletes. Individuals with physical or cognitive impairments were excluded. Participants were volunteers residing in Gaziantep, Turkey, who responded to a research advertisement and included students, as well as public and private sector employees. Ethical approval was obtained from the Non-Interventional Clinical Research Ethics Committee of Gaziantep Islamic University of Science and Technology (2024/402.37.03). The study was registered as a clinical trial (NCT06314347). Written informed consent was obtained from all participants. Anthropometric (height, weight, and body mass index [BMI]) and sociodemographic data were collected. The sample size was calculated using the Kasiulevicius V. formula (*n* = Nt2pq/d2(*N* − 1) + t2pq), and a target of 1,000 participants was determined [14].

### Figure of 8 walk test (F8WT)

The F8WT is a standardized clinical assessment used to evaluate advanced walking performance required in daily life. Two cones were placed 1.5 m apart, with participants standing midway between them. At the start of the test, the participant walked around the first cone as quickly and safely as possible, then continued around the second cone, forming a figure-of-eight trajectory. The test was repeated 3 times, and the mean time was recorded [10]. The validity and reliability of the F8WT have previously been demonstrated in populations with stroke, multiple sclerosis, and knee prosthesis [11–13].

### Data analysis

Descriptive statistics were reported as mean ± standard deviation or median (minimum–maximum) and percentiles for continuous variables, and as frequencies and percentages for categorical variables. Normality of distribution was assessed using the Kolmogorov–Smirnov test. To generate gender- and age-specific reference tables for the F8WT, participants were categorized into six age groups: < 20, 21–30, 31–40, 41–50, 51–60, and > 60 years. Typically, the 25th and 75th percentiles are used as the lower and upper cut-off values in a dataset. In studies on normative or reference values, scores between the 25th and 75th percentiles are generally considered “normal” or “average” for the construct under investigation [15]. Accordingly, F8WT performance below the 25th percentile was classified as “poor,” performance between the 25th and 75th percentiles as “moderate,” and performance above the 75th percentile as “good.” Associations with sociodemographic characteristics were examined using Pearson correlation analysis. Correlation strength was defined as low (0–0.49), moderate (0.50–0.69), high (0.70–0.79), and excellent (0.80–1.00). Independent t-tests were used to assess gender differences in anthropometric variables. A *p*-value of < 0.05 was considered statistically significant. All statistical analyses were performed using IBM SPSS Statistics (Version 20.0, IBM Corp., Armonk, NY, USA).

## Results

The mean age of the participants was 28.37 ± 13.29 years. Of the total sample, 279 (24.4%) were male and 866 (75.6%) were female. The mean height was 166.09 ± 0.86 cm, mean body weight was 67.16 ± 15.17 kg, and mean BMI was 24.30 ± 4.99 kg/m^2^. Sociodemographic and anthropometric characteristics by age group are presented in Table 1. Table 2 displays the F8WT results stratified by gender and age group, while Table 3 compares anthropometric measurements between males and females.

**Table 1:** General characteristics of the participants (*n* = 1145)

Variable	18–20 years (*n* = 324)	21–30 years (*n* = 530)	31–40 years (*n* = 107)	41–50 years (*n* = 73)	51–60 years (*n* = 49)	> 60 years (*n* = 62)
	mean ± SD	mean ± SD	mean ± SD	mean ± SD	mean ± SD	mean ± SD
Age, years	19,30 ± 0,77	23,24 ± 2,66	35,01 ± 2,94	45,35 ± 2,78	54,97 ± 2,56	67,17 ± 5,98
Height, m	1,66 ± 0,08	1,66 ± 0,08	1,68 ± 0,09	1,66 ± 0,08	1,63 ± 0,08	1,59 ± 0,08
Weight, kg	62,5 ± 14,65	64,03 ± 13,24	76,45 ± 15,21	79,41 ± 12,66	77,81 ± 13,82	78,95 ± 12,48
BMI, kg/m^2^	22,41 ± 4,02	23,03 ± 3,78	26,97 ± 5,06	28,60 ± 4,57	29,36 ± 5,66	31,32 ± 5,02

*BMI* body mass ındeks

**Table 2:** Age-sex-reference values for F8WT

	Minimum	25th percentile	50th percentile	Mean ± SD	Median	75th percentile	95th percentile	Maximum	*t*	*p*
18–20 years										
Male (*n* = 68)	3.06	3.78	4.21	4,49 ± 0,97	4.21	4.97	6.36	8.00	−0,718	0,473
Female (*n* = 256)	2.89	4.13	4.39	4,57 ± 0,75	4.39	4.90	6.00	8.63		
Total (*n* = 324)	2.89	4.05	4.37	4,55 ± 0,80	4.37	4.90	6.12	8.63		
21–30 years										
Male (*n* = 109)	3.12	3.99	4.46	5,01 ± 1,50	4.46	5.97	7.83	10.34	1,567	0,118
Female (*n* = 421)	2.41	4.06	4.51	4,80 ± 1,16	4.51	5.25	7.56	10.72		
Total (*n* = 530)	2.41	4.02	4.50	4,84 ± 1,24	4.50	5.32	7.57	10.72		
31–40 years										
Male (*n* = 40)	3.51	4.56	5.42	5,94 ± 1,70	5.42	7.81	8.72	9.34	0,874	0,384
Female (*n* = 67)	3.60	4.46	5.28	5,66 ± 1,54	5.28	6.25	9.10	10.59		
Total (*n* = 107)	3.51	4.47	5.28	5,77 ± 1,60	5.28	6.72	8.92	10.59		
41–50 years										
Male (*n* = 29)	3.47	4.30	5.10	5,90 ± 2,04	5.10	7.62	10.33	10.78	−1,454	0,150
Female (*n* = 44)	4.02	5.10	6.10	6,61 ± 2,03	6.10	7.40	10.60	13.63		
Total (*n* = 73)	3.47	4.67	5.84	6,32 ± 2,05	5.84	7.48	10.47	13.63		
51–60 years										
Male (*n* = 17)	4.41	5.54	7.30	7,98 ± 3,02	7.30	9.45	-	17.13	1,261	0,213
Female (*n* = 32)	3.41	5.05	6.54	7,00 ± 2,31	6.54	8.82	11.81	12.40		
Total (*n* = 49)	3.41	5.38	6.84	7,34 ± 2,59	6.84	9.15	11.95	17.13		
> 60 years										
Male (*n* = 16)	4.11	4.57	6.01	7,97 ± 5,00	6.01	9.36	-	22.31	−0,104	0,918
Female (*n* = 46)	4.94	6.19	7.16	8,07 ± 2,74	7.16	9.08	15.07	16.06		
Total (*n* = 62)	4.11	5.83	7.04	8,04 ± 3,42	7.04	9.21	15.65	22.31		
All										
Male (*n* = 279)	3.06	4.05	4.74	5,46 ± 2,22	4.74	6.21	9.34	22.31	2,538	0,011*
Female (*n* = 866)	2.41	4.17	4.63	5,15 ± 1,63	4.63	5.55	8.43	16.06		
Total (*n* = 1145)	2.41	4.14	4.65	5,22 ± 1,79	4.65	5.74	8.65	22.31		

*İndependent samples t test

**Table 3:** Sex differences in the anthropometric parameters

Variables	Male (*n* = 279) mean ± SD	Female (*n* = 866) mean ± SD	*t*	*p*
Age, years	31,12 ± 14,10	27,49 ± 12,91	3,995	0,000*
Height, m	1,76 ± 0,074	1,62 ± 0,058	31,42	0,000*
Weight, kg	79,75 ± 14,51	63,10 ± 13,00	18,06	0,000*
BMI, kg/m^2^	25,61 ± 4,30	23,88 ± 5,13	5,083	0,000*

*BMI* body mass ındeks, *İndependent samples *t* test

No significant differences in F8WT performance were observed between males and females across individual age groups (*p* > 0.05). However, a statistically significant difference was found when the entire sample was considered (*p* = 0.011). Based on F8WT performance, the 25th, 25th–75th, and > 75th percentiles were classified as low, average, and high fall risk, respectively, for both men and women. For men, < 3.06 s indicated low fall risk, 3.06–9.34 s indicated average fall risk, and 9.34–22.31 s indicated high fall risk. For women, < 2.41 s indicated low fall risk, 2.41–8.43 s indicated average fall risk, and 8.43–16.06 s indicated high fall risk (Table 2).

Statistically significant differences were observed between males and females in terms of age, height, body weight, and BMI (*p* < 0.01) (Table 3). Table 4 presents correlations between F8WT performance and sociodemographic as well as anthropometric parameters by age group. In the overall sample, a moderate positive correlation was found between F8WT and age (r = 0.575, *p* < 0.001). Additionally, low correlations were observed between F8WT and height (negative, r = –0.145, *p* < 0.001), body weight (positive, r = 0.242, *p* < 0.001), and BMI (positive, r = 0.359, *p* < 0.001) (Table 4).

**Table 4:** Correlation between F8WT and socio demographic and anthropometric parameters according to age groups

F8WT	Correlation (r)	*p*
18–20 years		
Age, years	−0,115	0,038*
Height, m	−0,113	0,041*
Weight, kg	−0,063	0,256
BMI, kg/m^2^	−0,017	0,765
21–30 years		
Age, years	0,287	0,000*
Height, m	−0,026	0,545
Weight, kg	0,081	0,062
BMI, kg/m^2^	0,011	0,530
31–40 years		
Age, years	0,065	0,503
Height, m	0,004	0,966
Weight, kg	0,154	0,113
BMI, kg/m^2^	0,185	0,056
41–50 years		
Age, years	0,270	0,021*
Height, m	−0,254	0,030
Weight, kg	0,402	0,000*
BMI, kg/m^2^	0,573	0,000*
51–60 years		
Age, years	−0,016	0,915
Height, m	−0,055	0,709
Weight, kg	−0,033	0,820
BMI, kg/m^2^	−0,004	0,981
> 60 years		
Age, years	0,659	0,000*
Height, m	−0,161	0,211
Weight, kg	−0,126	0,330
BMI, kg/m^2^	−0,028	0,832
All		
Age, years	0,575	0,000*
Height, m	−0,145	0,000*
Weight, kg	0,242	0,000*
BMI, kg/m^2^	0,359	0,000*

*BMI* body mass ındeks, *Pearson correlation

## Discussion

This study established reference values for the F8WT in healthy adults and examined the relationship between test performance and sociodemographic as well as anthropometric variables. The findings demonstrated a moderate positive correlation between F8WT time and age, a slight negative correlation with height, and slight positive correlations with body weight and BMI. These results suggest that F8WT performance, as reflected by longer completion times, declines markedly with increasing age, and that higher body weight and BMI are associated with poorer performance on the same measure. The absence of significant gender differences indicates that the F8WT is a reliable and applicable tool for assessing functional mobility in both men and women. Furthermore, the percentile values derived from this study provide clinicians and researchers with a practical framework for classifying fall risk.

In the present study, both weight and BMI were observed to increase with age. According to the World Health Organization, individuals aged 18–30 are generally classified as normal weight, those aged 31–60 as overweight (pre-obese), and those aged 60 and above as obese. National data from the Turkish Statistical Institute (Turkey Health Survey, 2022) indicate BMI ranges of 18.5–30.9 for adults aged 18–50 years and 40.6–48.2 for those over 50 years [16].

Interestingly, the BMI values in our sample were lower than those reported nationwide, which may be explained by the inclusion of only healthy individuals. The BMI findings are also consistent with studies validating the F8WT in different clinical populations, including research by Barker et al. on patients with knee replacements, Kırmacı et al. on individuals with multiple sclerosis, and Söke et al. on patients with Parkinson’s disease [10, 12, 13]. Similarly, Kong et al. [17] reported normal BMI values in elderly populations, and Wong et al. [11] found similar results in post-stroke patients.

The age-related increase in BMI is commonly attributed to reduced physical activity, changes in dietary habits, the presence of chronic diseases, and degenerative alterations in the musculoskeletal system [18, 19]. Age has also been suggested to influence gait characteristics [20]. In our study, mean F8WT times increased significantly with age, rising from 4.55 ± 0.80 s in the 18–20 age group to 8.04 ± 3.42 s in participants over 60 years. This finding highlights declines in motor performance, agility, and postural stability associated with aging. Previous reports of age-related mobility limitations by Bohannon and Andrews, as well as Karabulut et al., are in line with these findings [2, 21]. Similarly, our results are largely consistent with the normative values reported by Jude and Muskaan for Indian adults aged 60–69 years [22].

Although no significant gender differences in F8WT times were observed, men tended to perform the test more slowly than women. This may be explained by greater height and body mass in men, which can negatively affect gait stability during balance-demanding activities such as directional changes [8]. This highlights the ecological validity of the F8WT, which simulates real-life motor demands such as turning, agility, and directional adjustments, and demonstrates particular sensitivity in older populations.

A key finding of this study was the significant positive correlation between age and F8WT performance. This relationship likely reflects aging-related declines in muscle strength, joint mobility, balance control, and walking speed [23]. Longer walking times among older adults have been widely reported in the literatüre [24, 25]. In particular, the strong correlation between age and F8WT observed in participants over 60 years supports the marked deterioration of functional mobility with advancing age.

The negative association between height and F8WT suggests that taller individuals may walk faster due to longer stride length, a finding supported by earlier studies [26, 27]. However, the lack of significance in certain age groups (e.g., 31–40 years) indicates that biomechanical advantages may be counteracted by age-related declines in muscle strength, balance, or motor control.

Correlations between body weight, BMI, and F8WT were strongest in the 41–50 age group, suggesting that increasing weight during middle age has a particularly adverse effect on walking performance. Previous research has shown that obesity contributes to impaired balance control, excessive mechanical loading of the lower extremities, and reduced functional capacity [28, 29]. Obese individuals have also been reported to experience slower walking speed, reduced endurance, and decreased performance in activities of daily living [30]. Our findings are consistent with this evidence, indicating that weight-related gait impairments become especially pronounced in midlife.

Interestingly, a negative correlation between F8WT and both age and height was observed in the 18–20 age group. This may be explained by ongoing growth and developmental processes, as well as variability in physical fitness during this stage. High levels of physical activity, muscle strength, and balance control in this age group likely minimize age-related influences, making anthropometric differences more apparent.

This study has some limitations. First, the study was conducted exclusively in healthy adults; therefore, the reference values presented should be interpreted within this population and may not be directly applicable to individuals with chronic or neurological conditions. Second, the uneven distribution of participants across age groups may have influenced the statistical power of subgroup analyses.

## Conclusion

This study established normative reference values ​​for the F8WT in healthy adults, demonstrating its relationship to age, height, weight, and BMI. The findings indicate a significant decrease in walking performance with increasing age, and that increases in body weight and BMI negatively impact test performance. The F8WT’s age-sensitive assessment tool underscores the clinical importance of establishing age-specific normative values. These reference data may be used to support the classification of functional mobility and fall risk and to inform functional assessment and health screening in healthy adults.

## Data Availability

The data that support the findings of this study are available from the corresponding author upon reasonable request.
